# Using cryopreserved allogeneic pericardium to repair congenital heart defects in children

**DOI:** 10.1007/s10561-023-10089-x

**Published:** 2023-10-04

**Authors:** Mariusz Birbach, Maciej Fedorowicz, Ewa M. Gałkowska, Agnieszka Powirska, Michał Kozłowski, Krzysztof Mozol, Aleksandra Wasiak, Bohdan Maruszewski, Andrzej Kansy

**Affiliations:** 1 Department of Pediatric Cardiothoracic Surgery, The Children’s Memorial Health Institute, Aleja Dzieci Polskich 20, 04-730 Warsaw, Poland; 2 Allograft Heart Valve Cryobank, The Children’s Memorial Health Institute, Aleja Dzieci Polskich 20, 04-730 Warsaw, Poland

**Keywords:** Pericardium, Cryopreservation, Tissue banking, Cardiac surgery, Children

## Abstract

Patches prepared from autologous, allogeneic, or xenogeneic tissues are widely used in the repair of congenital heart defects in children. Since 2002, cryopreserved allogeneic pericardial patches have been prepared in our institution as an alternative to commercially available patches. This study retrospectively reviewed donor and patient data concerning cryopreservation time and the clinical use of the pericardium in 382 children who were operated on at a single center between 2004 and 2021. There were 177 donors: 98 males and 79 females. The median donor age was 13 years (range: 1 month to 53 years) and the median cryopreservation time was 72 days (range: 3–685). There were 382 pediatric patients: 224 males and 158 females. The median patient age was 1 month (range: 3 days to 17.8 years). The patches were used for primary surgeries in 228 patients and for reoperations in 154. The patches were implanted into the right heart or venous circulation in 209 patients, the left heart or arterial circulation in 246 patients, and both sides of the circulatory system in 73. Extracardiac patch implantation was performed in 339 patients, intracardiac in 79 patients, and both intracardiac and extracardiac in 36 patients. Our study presents a single-center experience in the use of cryopreserved allogeneic pericardium. The pericardium can be used on the systemic and pulmonary sides of the circulatory system, in either extracardiac or intracardiac positions. However, there is no uniform strategy for selecting the “patch of choice” for correcting congenital heart defects in children, especially since there are few studies comparing several types of patches.

## Introduction

Patches prepared from autologous, allogeneic, or xenogeneic tissues—including pericardium—are widely used in congenital heart defect repair in children. The pericardium consists of two components: parietal and visceral. The parietal pericardium is composed of an outer layer, called fibrosa, which is lined by pericardial mesothelial cells forming a serosa (Rodriguez and Carmela 2017). A comparison of allogeneic and autogenous pericardium use on an animal model was described as early as 1968 (Clarke et al. 1968).

Bioprosthetic heart valves which are composed of xenogeneic pericardium have been widely used in adult cardiac surgery for many years, since Ionescu’s pericardial valve concept in 1971 (Ohri et al. 2021). The clinical use of cryopreserved human pericardium is less common, though there are a few individual tissue banks in Europe where cryopreserved pericardia are prepared. The interest in the clinical use of such tissue to repair congenital heart defects in children is even less prevalent. This may be due to the commercial availability of xenogeneic pericardial patches at competitive costs and the limited number of studies comparing various types of patches.

In recent years some papers have been published on the decellularization of the pericardium, suggesting a conceivable way forward for future preparation of human pericardial patches (Mirsadraee et al. 2007, Neethling et al. 2014; Vinci et al 2013). However, clinical data on the utilization of such pericardium in humans are not yet available, let alone the surgical use of an allogeneic cryopreserved pericardium prepared in a cardiovascular tissue bank.

## Methods

The pericardium was harvested, processed, decontaminated, cryopreserved, and thawed according to the standard operating procedures following the national requirements and implemented in our cryobank. The pericardium was harvested only from multiorgan donors along with the heart, which was taken to prepare valved allografts. In some cases, only the pericardium was harvested.

The donor suitability criteria were determined based on information obtained from as many available sources as possible, taking into account medical documentation and history, the donor’s social behavior and age, and screening tests for HIV 1&2, HCV, HBV, syphilis, Sars-CoV-2, CMV, and toxoplasmosis. The age limit for donors was set at 55 years.

After harvesting, the pericardium—alone, in one or a few small pieces, or together with the retrieved heart—was flushed and placed in a double sterile bag in Ringer’s solution at a temperature of 4 °C. The pericardium was then placed in a transport bag with cooling pads. A sample of the donor’s blood was collected for serological evaluation. The donor’s serum is stored in our bank at − 70 °C for 30 years from the date of implantation of the pericardial patch, which is mandatory according to national requirements.

The pericardium was prepared under sterile conditions in a laminar flow cabinet with Class A air cleanliness. Epipericardial fat residue, fibrotic tissue, and remnants of the parietal pleura were dissected as necessary. Pericardial patches measuring 30 × 40 mm and 40 × 50 mm or larger were prepared, depending on the size of the pericardium retrieved. The pericardium was decontaminated for 24 h at room temperature in an antibiotic nutrient medium composed of piperacillin/tazobactam, vancomycin, gentamicin, and nystatin in 200 ml of RPMI 1640 (Roswell Park Memorial Institute, USA). Bacteriological tests of the pericardium and the medium were carried out before and after decontamination preceding cryopreservation, and before implantation of the thawed pericardium. From 1995 to 2018 we used two methods of bacteriological examination for cardiovascular tissues: the conventional method and the automated microbial detection system, BacT/Alert (bioMérieux, FR). Since that time, we have been using the Bactec FX200 (Becton Dickinson and Company, USA) as our automated system.

The conventional method was carried out in sugar broth at 37 °C within 7 days for aerobes, in Schaedler broth for 7 days for anaerobes (in the past, we have used thioglycolate medium in an increased CO2 concentration at 37 °C for 4–7 days), and on Sabouraud medium at 25 °C for 30 days for fungi. For the automated detection system, we prepared a homogenate of cardiac tissue retrieved along with the pericardium. We did not prepare a homogenate of the pericardium. In the automated method, the homogenate was incubated in fluid media with antibiotic inhibitors: Bactec™ Lytic/10, Anaerobic/F, and Bactec™ Plus Aerobic/F (Becton Dickinson). Incubation was carried out for 5 days to determine the presence of aerobes and anaerobes and for 10 days in the case of fungi.

The decontaminated pericardial patches were placed in double sterile EVA bags (Macopharma, France) filled with 10% DMSO (≥ 99.5%; Reagent Plus®, Sigma-Aldrich) in RPMI 1640 and cryopreserved. Cryopreservation was performed using an IceCube (Sy-Lab, AT) computer freezer with a controlled freezing rate. The patches were then stored in tanks with liquid nitrogen vapor below − 150 °C for a maximum of 5 years.

For the thawing procedure, the pericardium was taken out of the liquid nitrogen tank and left for 10 min at room temperature, as we routinely do with valved allografts, to avoid rapid thawing and possible damage to the tissue (Wassenaar et al. 1995). The pericardium was then placed in a dry water bath (Plasmatherm DTM/DTMII, Barkey, Germany) at 37 °C for 10 to 15 min. The EVA bag was opened in the laminar flow cabinet and the pericardial patch was rinsed in 100 ml of RPMI 1640 with 5% DMSO (at 2–8 °C), followed by flushing with another 100 ml of RPMI. The pericardium was then placed in a double sterile container (Freezable Tissue Storage Kit, Medford Products Ltd) and delivered to the operating room.

### Statistical analysis

Data compilation consisted of grouping patients by procedure: primary surgery, elective reoperation in a multistage treatment, and other reoperation. The patients operated on in the first month of life were in a separate sub-group, accounting for more than 44% of all operations and more than 74% of primary surgeries.

The incidence of surgical access from the right heart, left heart, or both were counted from a binomial distribution. The incidence rates were compared using Student’s t‑test, with 0.05 taken as the level of statistical significance for the differences. The analysis of intracardiac, extracardiac, and mixed operations and any other incidence rate comparisons were done similarly.

## Results

We have been preparing cryopreserved allogeneic pericardia in our cryobank since 2002. Of the 484 pericardial patches prepared, 473 were distributed for clinical use between 2002 and 2021. For this study, we retrospectively reviewed donor and patient data regarding the cryopreservation time and clinical use of cryopreserved allogeneic pericardia in 382 children operated on in our institution for congenital heart defects.

All pericardial patches were harvested from multiorgan donors. The discard rate of this pericardium was insignificant. There were 177 donors: 98 males and 79 females. The median donor age was 13 years (range: 1 month to 53 years) and the median cryopreservation time was 72 days (range: 3–685). Our patient cohort for this study included 382 pediatric patients: 224 males and 158 females. The median age was 1 month (range: 3 days to 17.8 years). The largest group of cryopreserved pericardial patch recipients for primary operations were patients up to 1 month old. In our entire group of 382 pediatric patients, 228 primary surgeries were performed; the remaining 154 were reoperations, half as a part of multistage treatment of congenital heart defects. The primary surgeries were mostly performed on patients less than 1 month old, whereas the majority of the elective multistage-treatment reoperations were in patients less than 1 year old. The other reoperations were mostly performed in children over 1 year of age. Sixteen patients from the primary surgery group who required subsequent surgery were in the reoperation group. The reoperations in this group were not patch related in all but one patient who developed re-coarctation of the aorta and stenosis of the aortic arch.

**Fig. 1:**
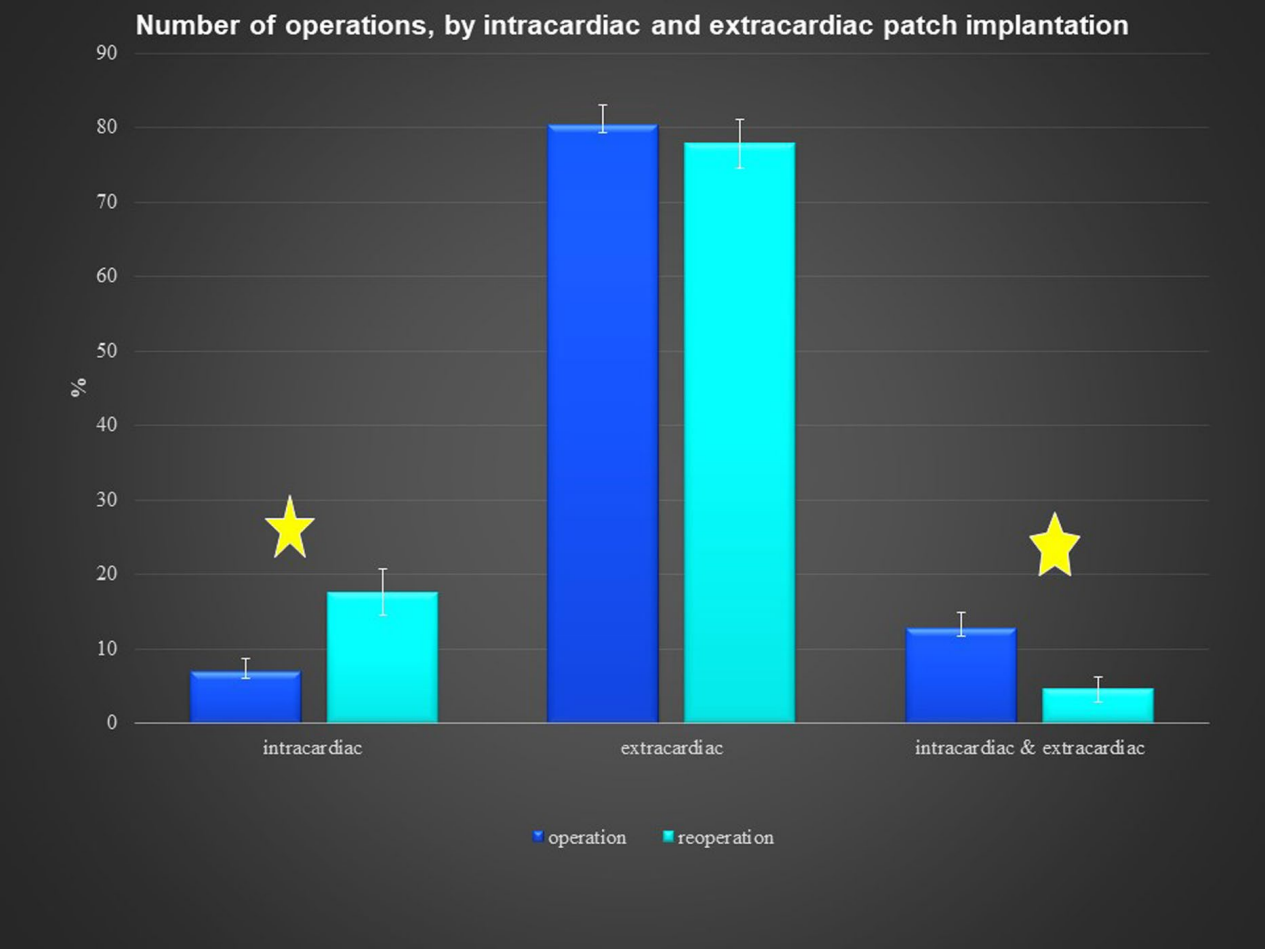
Number of operations, by intracardiac and extracardiac patch implantation

**Fig. 2:**
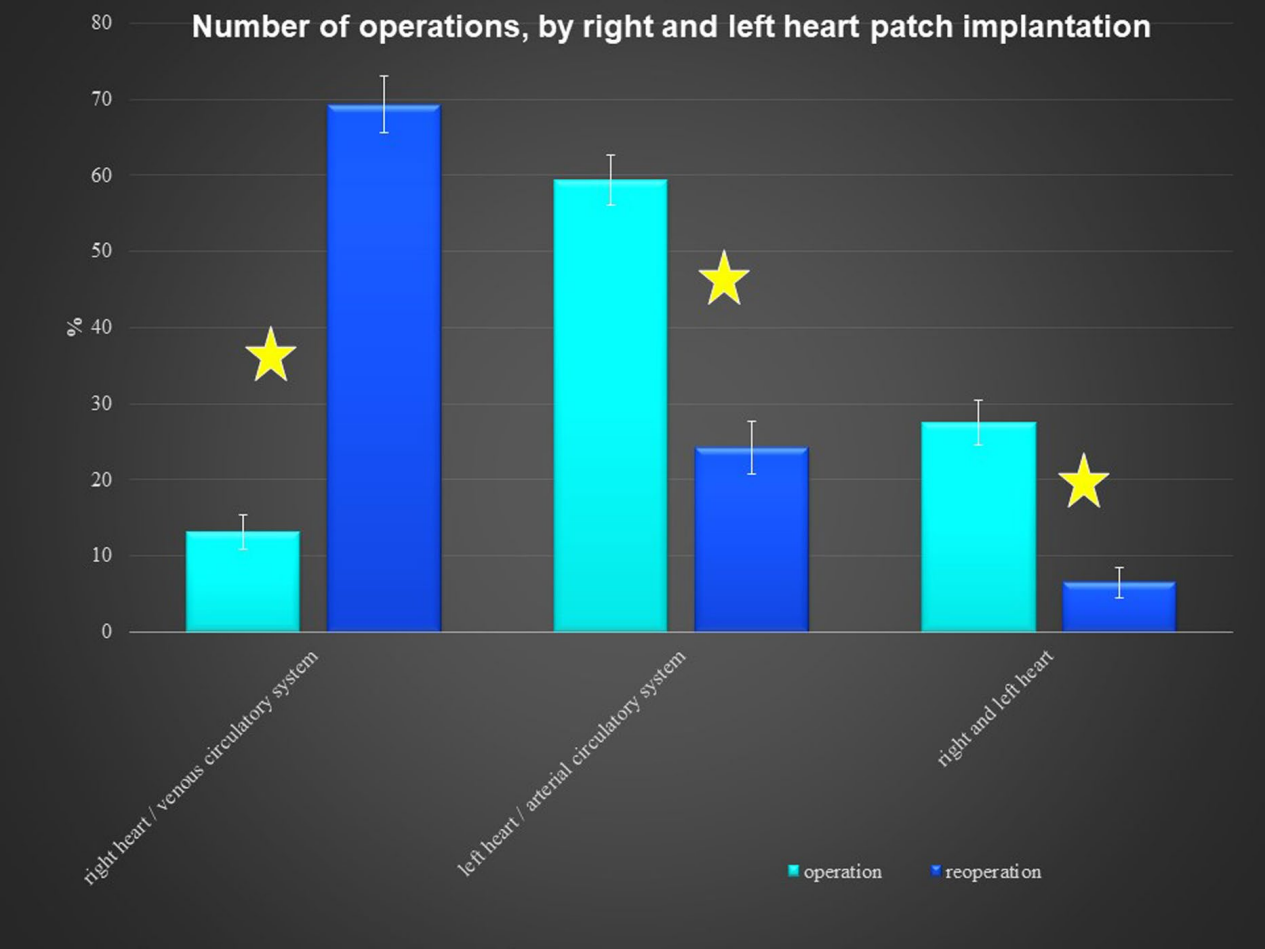
Number of operations, by right and left heart patch implantation

**Fig. 3:**
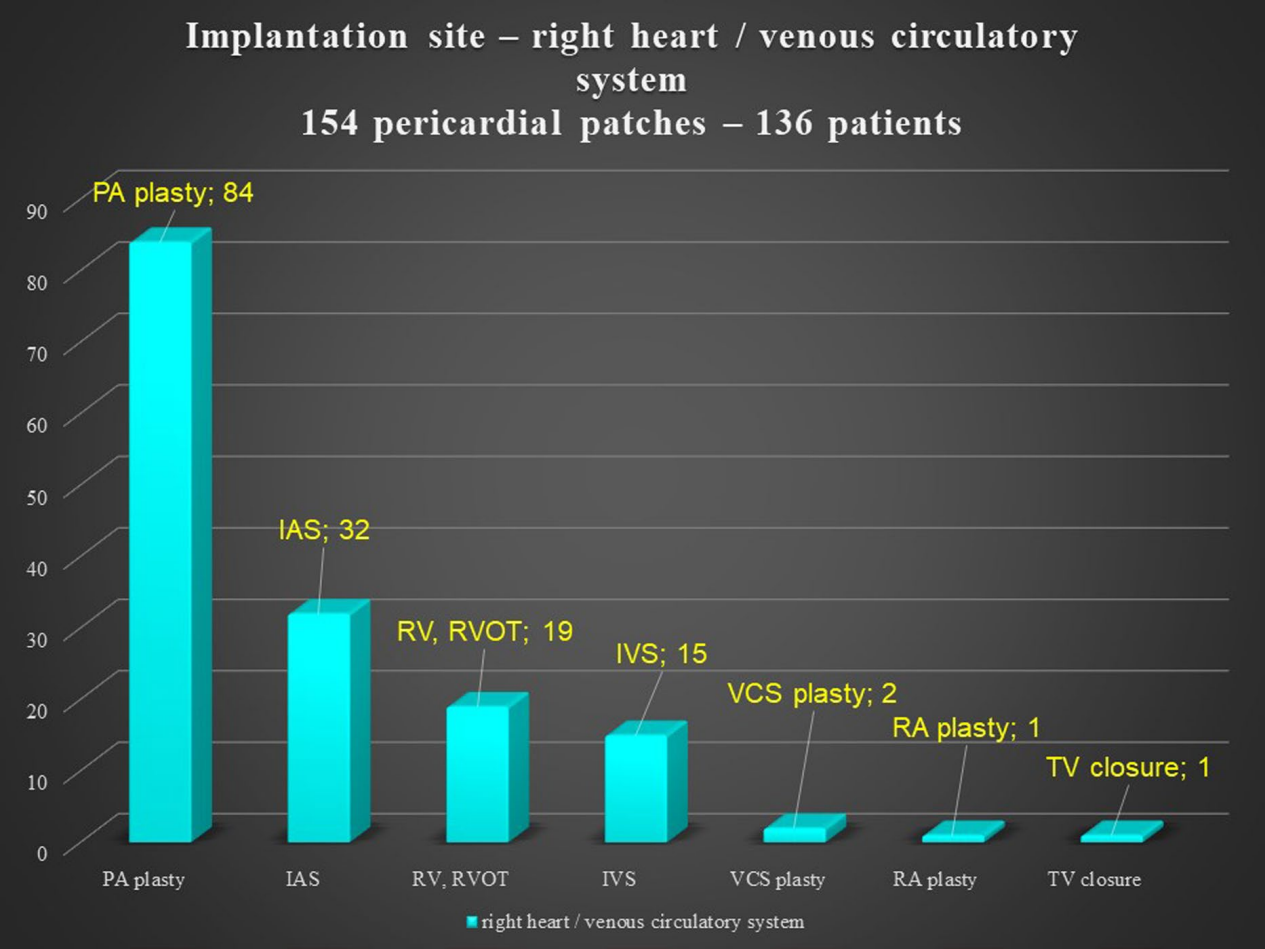
Right heart / venous circulatory system implantation site. PA: pulmonary artery; IAS: interatrial septum; RV: right ventricle; RVOT: right ventricle outflow tract; IVS: interventricular septum; VCS: vena cava superior; RA: right atrium; TV: tricuspid valve

**Fig. 4:**
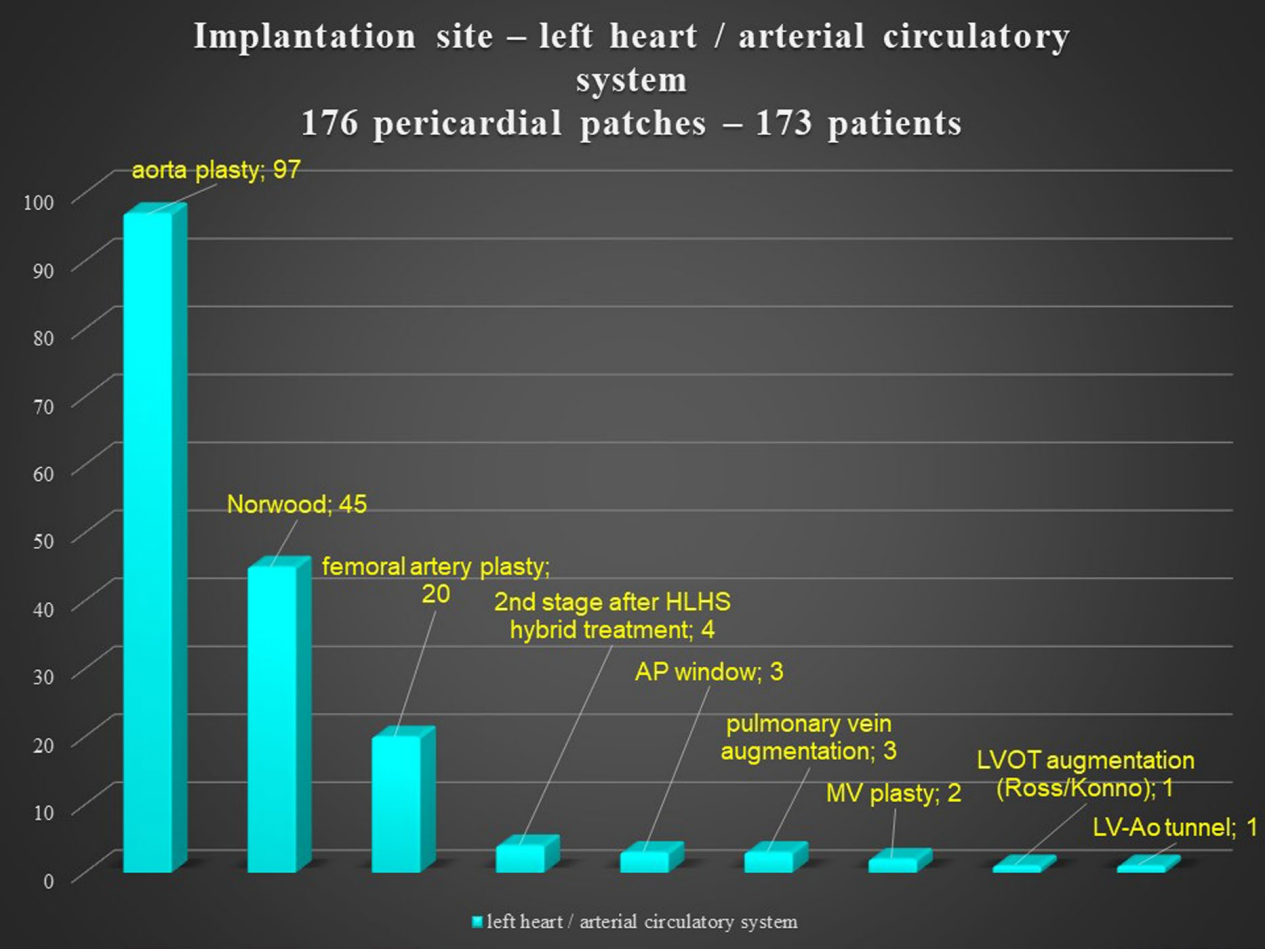
Left heart / arterial circulatory system implantation site. HLHS: hypoplastic left heart syndrome; AP window: aortopulmonary window; MV: mitral valve; LVOT: left ventricle outflow tract; LV-Ao tunnel: left ventricle – aorta tunnel

**Fig. 5:**
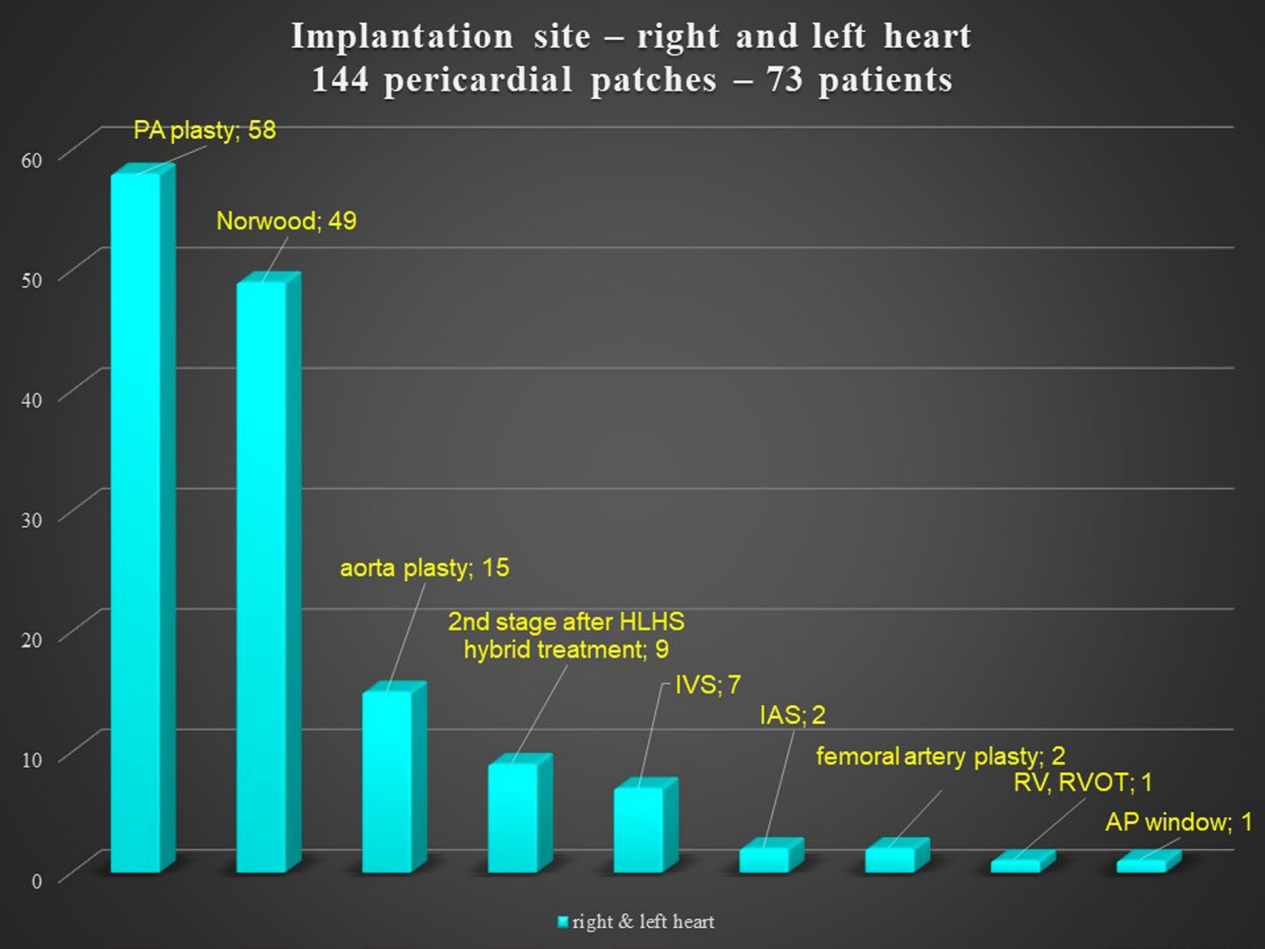
Right and left heart implantation site. PA: pulmonary artery; HLHS: hypoplastic left heart syndrome; IVS: interventricular septum; IAS: interatrial septum; RV: right ventricle: RVOT: right ventricle outflow tract, AP window: aortopulmonary window

**Fig. 6:**
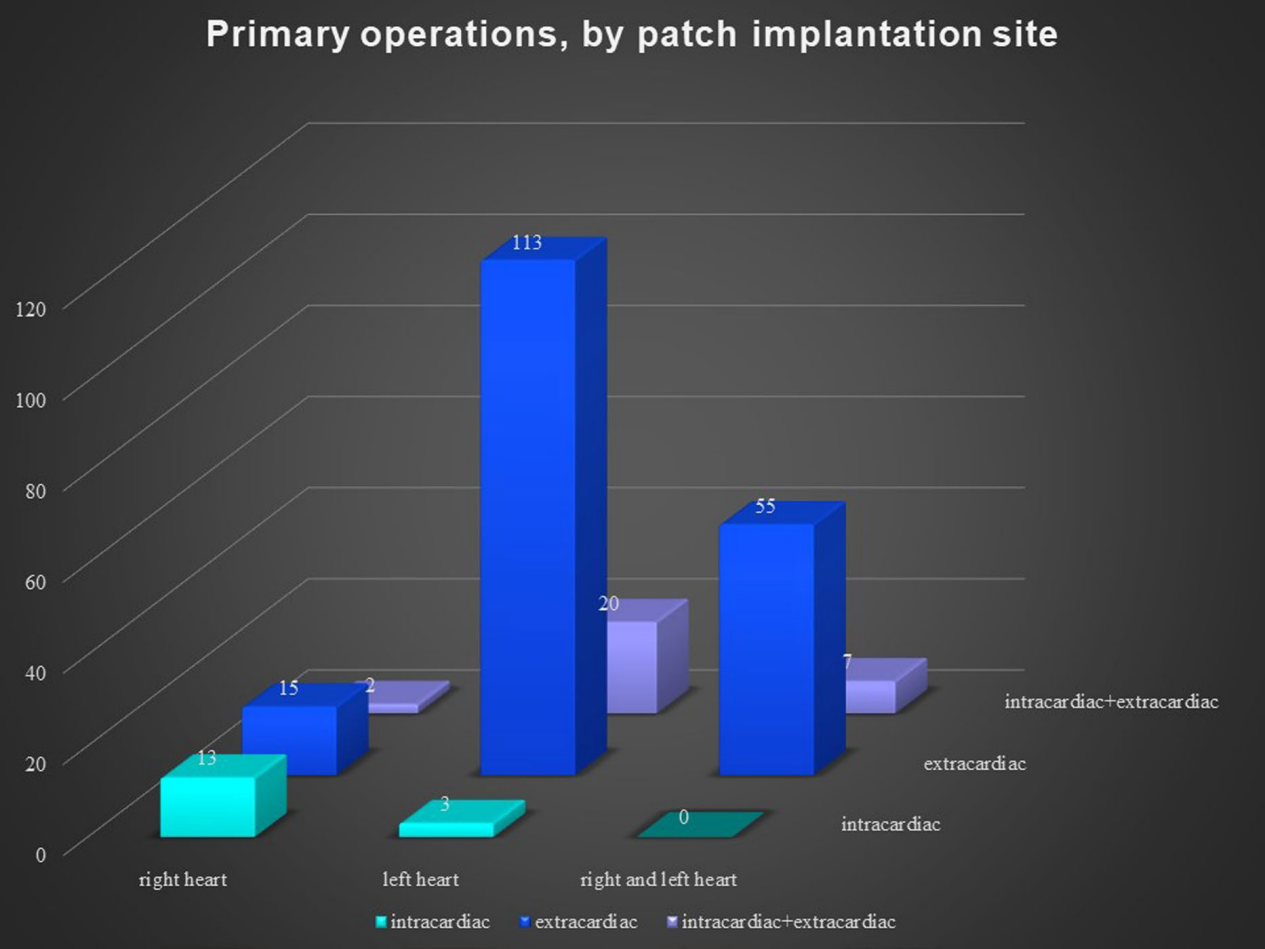
Primary operations, by patch implantation site

**Fig. 7:**
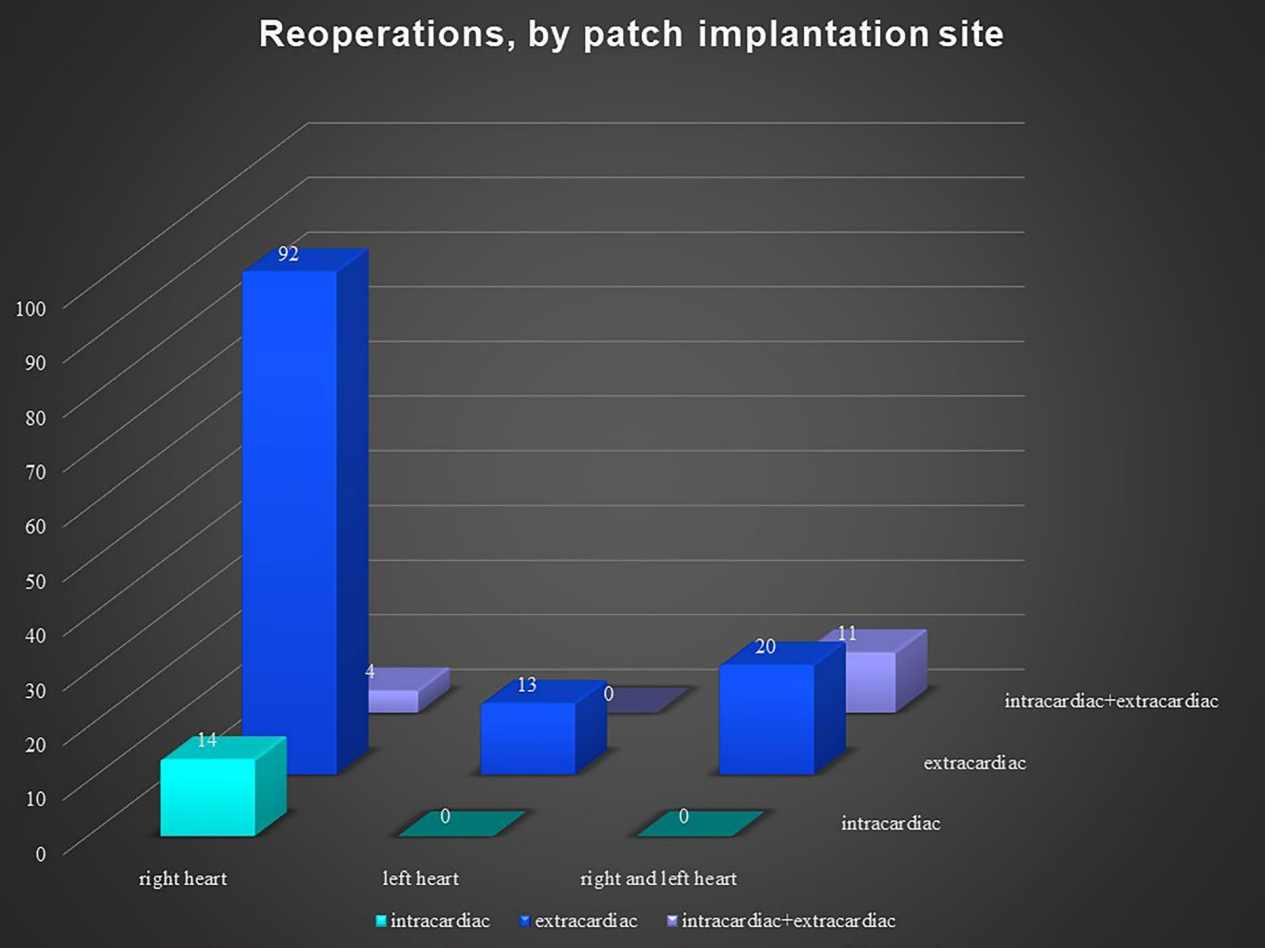
Reoperations, by patch implantation site

There was no significant difference according to sex in the reoperation group, but the primary operation group had more males. The pericardial patches were implanted in the extracardiac position in 339 patients, in the intracardiac position in 79 patients, and in both positions in 36 patients. In 8 patients, we used 2 pericardial patches from the same donor. This small group represented 7 reoperations and 1 primary operation. There was no difference between the operation and reoperation groups regarding the extracardiac position of the patch, though there was a significant difference between the operation and reoperation groups in intracardiac and dual placement of the patch (Fig. 1). There were 209 implantations in the right heart and venous circulatory system, 246 implantations in the left heart and arterial circulatory system, and 73 implantations in both the right and left heart. There was a significant difference between the primary operation and reoperation groups regarding right and left heart patch implantation: reoperations were more prevalent with right heart placement of the patch, while primary operations were predominant with left heart placement (Fig. 2). On the left side, the pericardial patches were used most often for aorta plasty and the Norwood procedure (Fig. 3), while on the right heart, the pericardial patches were most often used for pulmonary artery plasty (Fig. 4). In the group of 73 patients with implantation on both sides, the patch was most often used for pulmonary artery plasty and the Norwood procedure (Fig. 5). Overall, for primary operations, the patch was used most often on the left heart and in the extracardiac position (Fig. 6), while for reoperations the patch was most often implanted on the right heart and in the extracardiac position (Fig. 7). Statistically significant differences were found in the incidence of right and left heart patch implantation and of intracardiac and extracardiac patch implantation for primary operation and reoperation.

## Discussion

We have been preparing cryopreserved allogeneic pericardia in our cryobank since 2002. The limited number of pulmonary valved allografts which may serve as a source for patches, the limited number of commercially available xenogeneic pericardial patches, and the limited number or lack of studies comparing different types of xenogeneic and allogeneic patches all influenced our decision to begin preparing such patches. In most situations, the patch of choice for primary heart operations—especially in small children—should be the autogenous pericardium. However, for reoperations, where there are many pericardial adhesions, the amount of available autogenous pericardia is limited.

In the past, we used autologous pericardia fixed in glutaraldehyde. This procedure changed when the regulations limiting the use of glutaraldehyde in operating rooms were implemented in our institution. The use of cryopreserved allogeneic pericardia which have not been treated in glutaraldehyde became one of the methods for choosing a patch for heart operations in children in our hospital.

Fixation of cardiovascular tissues in glutaraldehyde is a well-known method for preserving xenogeneic patches and valves, autologous pericardia, and cryopreserved allogeneic pericardia (Jayakrishnan and Jameela 1996, Gluck et al. 2022). Glutaraldehyde reduces the immunogenicity of the pericardium through cross-links between the matrix and soluble proteins, but it may lead to accelerated calcification (Mirsadraee et al. 2007; Laing et al. 2010) and shrinkage of the tissue. For these reasons, we used cryopreserved allogeneic pericardial patches for primary operations in our patients, even though autogenous pericardium was available.

Most of the patches were used in the extracardiac position on the right and left sides of the circulatory system, because the patches were more often used for plasty or augmentation of extracardiac structures, such as the aortic arch on the left side of the heart or the pulmonary arteries on the right side. There was a statistically significant difference between intracardiac placements of the patches (Fig. 1) used for reoperations. During reoperation, there is a limited amount of autogenous pericardium, which is especially needed for the closure of interatrial communication. Pulmonary artery plasty was the most performed procedure for reoperation on the right heart, and aortic arch plasty was the most performed procedure for operation on the left heart and both influenced statistical difference between the primary operation and reoperation groups (Fig. 2). Reoperations on the aortic arch in children have become less common since catheter-based cardiology procedures were implemented. Overall, during the primary operation, the patch was most often implanted in the left heart and in the extracardiac position, while for reoperations the patch was most often implanted in the right heart and in the extracardiac position as well.

The ideal material for a patch used in pediatric cardiac surgery should be durable, non-thrombogenic, non-immunogenic, and available in any size and should have growth potential (Waqanivavalagi et al. 2020). The following factors favored the extensive use of allogeneic pericardium in our patients. Cryopreserved pericardium is always available in our cryobank and is easy to harvest and prepare. Allogeneic pericardium is pliable and favorable to surgical handling, demonstrating its versatile use. On the other hand, it may be a bit thick when harvested from older donors, in which case it may not be the best option to use in neonates or infants in specific anatomical positions, such as pulmonary veins or arteries.

Compared to xenogeneic patches, the autologous pericardium is more flexible and flaccid, less rigid, and lays better on the tissue during suturing, just like autologous pericardium. Handling is very good for the surgeon except when the pericardium is from an adult donor. Then the pericardium can feel thickened when passed with a thin surgical needle. We did not observe the shrinkage of implanted pericardium during the next stage of surgery, nor calcification.

The question remains whether it is competitive to xenogeneic pericardial patches, since there is limited data comparing the two types of patches.

## Limitation of the study

This is a retrospective study on the clinical use of cryopreserved allogeneic pericardial patches which are not treated in glutaraldehyde. We did not compare this group to a group with implanted xenogeneic or autogenous patches which may or may not have been treated in glutaraldehyde. We do not present the follow-up of the patients, especially in terms of the different types of implanted patches and patch-related catheter interventions. However, the follow-up of patients from a specific group of Norwood procedures—where a cryopreserved allogeneic pericardial patch or allogeneic pulmonary allograft tissue was implanted—will be evaluated in this regard.

The patients from the primary surgery group who required reoperation without cryopreserved pericardia were not included in this study as well the patients from both reoperation groups who needed subsequent reoperation.

In conclusion, our study presents a single-center experience in the use of cryopreserved allogeneic pericardium to repair congenital heart defects in children as an alternative to synthetic and xenogeneic patches. The pericardium was used on both the systemic and pulmonary sides of the circulatory system, in either the extracardiac or intracardiac position. However, there is no uniform strategy for selecting the “patch of choice” for surgeries on congenital heart defects in children, especially since studies comparing several types of patches are very limited.

## Data Availability

The datasets for this study were collected and analyzed in Department of Cardiothoracic Surgery and Allograft Heart Valve Cryobank, Children’s Memorial Health Institute and may be available on reasonable request. The Institutional Bioethics Committee approved the study and individual consent for the study was waived. Upon admission to our hospital, all children's guardians sign consent for the use of blinded clinical data for scientific research. Due to the retrospective analysis of the data, separate parental consent was not required. Approval of the local bioethics committee for the study was obtained.
